# Neuron type classification in rat brain based on integrative convolutional and tree-based recurrent neural networks

**DOI:** 10.1038/s41598-021-86780-4

**Published:** 2021-03-31

**Authors:** Tielin Zhang, Yi Zeng, Yue Zhang, Xinhe Zhang, Mengting Shi, Likai Tang, Duzhen Zhang, Bo Xu

**Affiliations:** 1 Institute of Automation, Chinese Academy of Sciences, Beijing, China; 2 University of Chinese Academy of Sciences, Beijing, China; 3 Center for Excellence in Brain Science and Intelligence Technology, Chinese Academy of Sciences, Shanghai, China; 4 Electronics and Communication Engineering, Peking University, Beijing, China; 5 Department of Automation, Tsinghua University, Beijing, China

**Keywords:** Classification and taxonomy, Machine learning

## Abstract

The study of cellular complexity in the nervous system based on anatomy has shown more practical and objective advantages in morphology than other perspectives on molecular, physiological, and evolutionary aspects. However, morphology-based neuron type classification in the whole rat brain is challenging, given the significant number of neuron types, limited reconstructed neuron samples, and diverse data formats. Here, we report that different types of deep neural network modules may well process different kinds of features and that the integration of these submodules will show power on the representation and classification of neuron types. For SWC-format data, which are compressed but unstructured, we construct a tree-based recurrent neural network (Tree-RNN) module. For 2D or 3D slice-format data, which are structured but with large volumes of pixels, we construct a convolutional neural network (CNN) module. We also generate a virtually simulated dataset with two classes, reconstruct a CASIA rat-neuron dataset with 2.6 million neurons without labels, and select the NeuroMorpho-rat dataset with 35,000 neurons containing hierarchical labels. In the twelve-class classification task, the proposed model achieves state-of-the-art performance compared with other models, e.g., the CNN, RNN, and support vector machine based on hand-designed features.

## Introduction

It has been more than 100 years since Santiago Ramón y Cajal first proposed the neuron doctrine by staining sparse cell populations in 1906. After that, more research came and joined the ranks, which have promoted this research area's development. Currently, we have more elaborate and efficient equipment for a better understanding of the neuron doctrine from different scales, disciplines, and perspectives.

Like Mendeleev in 1869, who developed the periodic table, many researchers have made efforts to conduct neuron type classification and multi granularity neural identity measurements from different brain regions^1^. Many improvements have been achieved in neural classification based on the molecules, electrophysiology transcriptome, genome, biophysics, and morphology, to understand biological structures and functions better systematically and reproducibly. However, there is currently no broadly accepted and generally agreed-upon approach for neuron type classification until now.

For a finer neuron categorization, one smart way is to incorporate the features from different perspectives as much as possible. For example, Zeng et al. took advantage of quantitative features (e.g., structural, functional, and molecular criteria) measured from the same tissue^2,3^. However, limited by experimental procedures or experimental equipment, for most cases, only one or two specific criteria can be obtained from them. From the transcriptomics perspective, 23,822 cells were categorized into 133 cell types with the single-cell RNA sequencing method in the primary visual cortex and the anterior lateral motor cortex^4^. From the molecular criteria perspective, the relationships between molecular cell types and the morphological, physiological, and behavioral correlations measured by spatially resolved transcriptomic methods have been well identified^5^. In addition, the developmental and evolutionary definition of a cell type based on the molecular basis is also conducted within and between different species^6^. From the genome's perspective, the Ivy Glioblastoma Atlas is constructed based on different features of genomic alterations and gene expression patterns^7^. From the perspective of electrophysiology, the Allen Cell Types Database has generated 170 individual biophysically detailed neuron models and identified the distinctiveness of the intrinsic properties between subsets of these cells in the cerebral cortex^8^.

The prevailing schemes of neuron-type nomenclature contain the morphology definition, anatomical definition, molecular definition, physiological definition, evolutionary definition^6^, and developmental definition (multiple species comparison or lineage tracing on a larger time scale an animal). Hence, neuron types could be glutamatergic neurons, GABAergic neurons, parvalbumin-expressing neurons, somatostatin-expressing neurons, or glial cells. The study of cellular complexity in the nervous system based on anatomy shows more practical and objective advantages in morphology than other perspectives, such as the molecular, physiological, and evolutionary aspects. New progress in neuron staining, slicing, and reconstruction methods in mouse and rat brains^9–11^ have also contributed to neuronal morphology identification.

NeuroMorpho.org is one of the largest public neuronal morphology databases of digitally reconstructed neurons by species, brain regions, and cell types^12^ and currently contains 721 cell types, 112,244 neurons, and 317 brain regions. Hippocampome.org is another morphology dataset with detailed axonal and dendritic patterns for the mouse hippocampus, containing 122 neuron types categorized by integrating morphology and neurotransmitters, synaptic specificity, electrophysiology, and molecular biomarkers^13^. The 3D morphologies and somatic electrophysiological responses combine for 170 individual neuron identifications in the primary visual cortex^8^. Anatomical methods have also been well integrated with genomic methods for validating the neuronal cell types and their connections in multiple species in the BRAIN Initiative Cell Census Consortium^14^.

However, the exact class numbers of rat neurons from taxonomy are still unknown^15^. This dilemma possibly comes from two main reasons: one is that the definition of different types of neurons is still not clear; the other is that one specific neuron type usually contains many diverse subtypes^4^, making their identification difficult, especially for some inhibitory neurons.

Traditional methods depend mainly on specific hand-designed features (e.g., the size of the soma, the number of branches, the angle of bifurcation, the branch level, the branching order, the neuron length, the dendritic and axonal shapes, and branching patterns, and the spine density), which are labor-intensive and possibly failed for some neurons with very complex dendritic or axonal arbors. Hence, the neuron-type classification automatically is becoming more necessary and urgent for the further analysis of multiscale neural circuitry and the functional simulation of biological networks.

**Figure 1:**
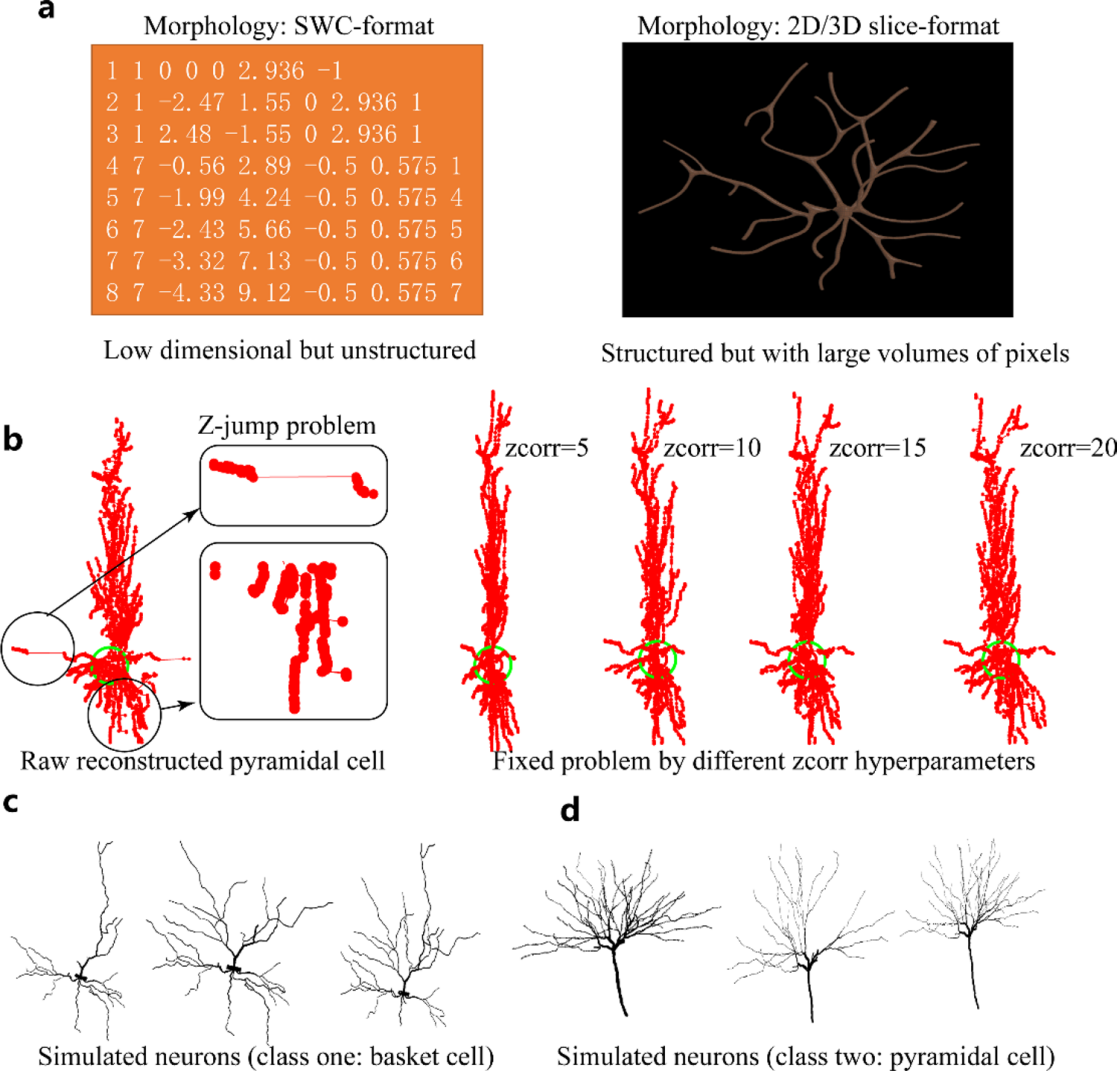
(**a**) Comparisons of the two main formats of neuronal morphology. (**b**) Morphological repairment of a single neuron by the Trees toolbox with different hyperparameters of the “zcorr” method. (**c**) Virtual simulated basket-type neuron samples containing different branch orders generated with Trees toolbox. (**d**) Generated pyramidal-type neurons with Trees toolbox.

The morphologies of neurons are usually described in two types of neuronal formats: one type is the SWC-format file; the other is the 2D- or 3D-image format, as shown in Fig. 1a. However, the SWC-format file is low dimensional and unstructured (e.g., compressed as the structure of the non-strict binary tree, and invariant with exchanging neighborhood structures under the same bifurcate; moreover, the node list length is closely connected with the sampling rate). The 3D-image format is structured but with too high information dimensions (e.g., fewer samples than the complexity of the morphology, similar local features, invariance to translation rotation, and large scale of diameters). This paper focuses on feature detection from different data formats and tries to find some practical solutions for neuron type classification by morphology.

Deep neural networks (DNNs) are powerful in both structured and unstructured information processing. They possess many different kinds of subtypes, for example, the feed-forward type convolutional neural network (CNN) or the loop-based recurrent neural network (RNN). These different types of architectures make DNNs powerful for both spatial and temporal information processing. For example, DNNs have been successfully applied to spatial information abstraction tasks (e.g., ImageNet classification^16–19^), sequential information prediction tasks (e.g., video-to-text transformation^20,21^), long-term planning tasks (e.g., go games^22^ and Atari 2600 games^23^), and so on. The development of neuroscience and machine learning has also contributed to the development of automatic neuron type classification and categorization^1^, including expert knowledge^24^, the Bayesian classifier^25^, Bayesian clustering^15^, K-means clustering^26^, affinity propagation clustering^27^, harmonic co-clustering with the L-measure^28^ and hierarchical clustering^29^.

In this paper, we obtain inspiration from the morphology of neurons, which are more similar to tree-type structures, and then construct an integrative DNN model that contains a Tree-RNN^30^ and ResNet CNN for a better neuron-type classification. We design a Tree-RNN for feature learning from the unstructured SWC-format file data and construct a ResNet CNN for feature detection from structured 2D neuron images. We combine these two models, i.e., integrating the high-level features (at the last full-connection layers for each model) together by concatenating two feature vectors together as a longer vector then classified it with a new three-layer neural network. The model is then verified on the virtually simulated dataset (with generated two-class neuron data, relatively simple), CASIA rat-brain dataset (with 2.6 million reconstructed neurons without labels), and standard NeuroMorpho-rat dataset (with 35,000 labeled neurons with two main classes and twelve subclasses downloaded from the NeuroMorpho website, relatively hard).

## Results

### Virtually simulated dataset

Usually, it is hard to identify which reason has caused the lousy performance of classification when both the complexity of datasets and the complexity of methodologies are not measured. Here, we try to lock the dataset's complexity first by generating some virtual simulated samples first and then testing them with the proposed classification methods. By making this effort, we can quickly filter out some designed but relatively weak classifiers.

Z-jump in the SWC-format file is a commonly occurred problem, especially for some samples without well tracing, where the values of z axes of some feature points jumped (Fig. 1b, left). Some post-processing methods such as smoothing or filtering can handle this type of problem at some scale. For example, a demo of automatic repair of the Z-jump problem (as shown in Fig. 1b, right) is given with the “zcorr” function from Trees toolbox^24^. Then, point resampling is performed on the SWC-format files to obtain a better point distribution. This process includes diameter alignment and morphology smoothing. Finally, we use the Trees toolbox^24^ for the virtual simulated sample generation. For example, the 2-class virtual simulated dataset is generated with different neuron properties. The 2D images, which can be considered the projection of raw 3D images, are also generated with different hyperparameters. In order to keep the training samples balanced, we also use this Trees toolbox to expand the dataset (including both SWC files and images) by generating similar samples (with little pruning and addition of samples in the target class, 90%) and carry out the transformation of images by rotating, zooming in or zooming out.

The virtually simulated dataset contains two basic types of neurons: the basket cell as shown in Fig. 1c and the pyramidal cell as shown in Fig. 1d. The source code is available at https://github.com/thomasaimondy/ treestoolbox/tree/master/casia. The two types of neurons have 500 samples for each class, with both the SWC-format and 2D-image-format data converted by the Trees toolbox.

### NeuroMorpho-rat dataset

**Figure 2:**
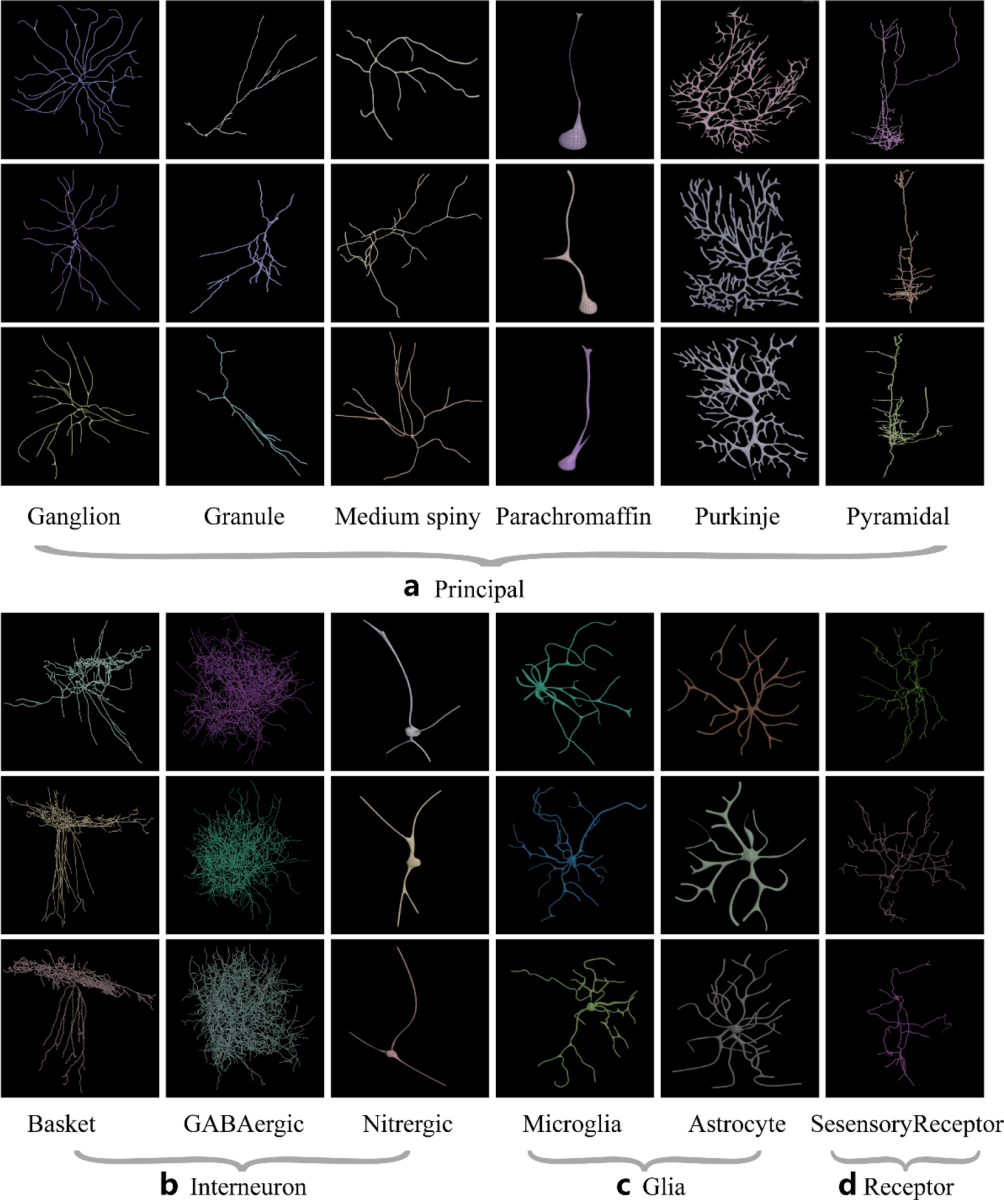
The neuronal morphologies from SWC-format data after repairing with Trees toolbox. The Houdini software generates the 3D model of neurons. Here, we show three examples for each class of neurons in the NeuroMorpho-rat dataset. (**a**) The six types of principal cells: ganglion, granule, medium spiny, parachromaffin, Purkinje, and pyramidal cells. (**b**) The three types of interneuron cells: basket, GABAergic, and nitrergic cells. (**c**) The two types of glial cells: microglia and astrocyte cells. (**d**) The 1 type of sensory receptor cell.

Several (approximately 20% by calculation) of the neuronal morphology files at NeuroMorpho.org present the Z-jump problem. The Trees toolbox has a “zcorr” function designed for SWC file repairment. Figure 2 shows neurons after being repaired and processed by the Houdini software based on different hyperparameters (e.g., “zcorr = 10”). The data contains two main classes (principle cell and interneuron cell) and 12 subclasses, including six types of principal cells (e.g., ganglion, granule, medium spiny, parachromaffin, Purkinje, and pyramidal cells) in Fig. 2a, three types of interneuron neurons (e.g., basket, GABAergic, and nitrergic) in Fig. 2b, two types of glial cells (e.g., microglia and astrocytes) in Fig. 2c and one type of sensory receptor cell in Fig. 2d. The number of selected rat samples (e.g., the rat neurons) with labels is 35,000.

### CASIA rat-neuron dataset

Most of the state-of-the-art single-cell reconstruction algorithms^10,11,31^ use the tracing-based method for the identification of neuron structures. However, it is time-consuming and difficult to achieve a full-brain-size reconstruction atlas in this way. The raw slice data in the CASIA rat-neuron dataset are from the lab of Qingming Luo^9^. Here, we reconstruct neurons with classification methods, which classifies (or separates) all of the biological structures from the background. The activation spreading method is then used for the single-cell morphology identification (e.g., SWC-format file). This method is efficient and can obtain 2.6 million neurons in a much shorter time, even though some connections with much longer distances are missing. The detailed introduction of biological tissue recognition, reconstruction, and segmentation will be further described in our next paper.

We use most of the well-preserved relatively local information (e.g., soma and the locally connected synapse located within 500 µm) as the essential information for neuron type classification. However, this dataset does not contain neuron labels. This paper uses it as the pretraining dataset before neuron classification of the labeled data (e.g., NeuroMorpho-rat dataset) in the CNN-based and RNN-based models.

The pretraining is commonly used in many DNN architectures. First, two DNN architectures with one ordinary and one mirror network are designed. Then, the two networks are connected for the information encoding and decoding. As an input image X, for example, the network encoded it into a Y with smaller dimensions and then decoded it back into X (represented as $\widehat{X}$), where the loss of network is designed as the minimal square error of X and $\widehat{X}$. We reconstruct both the CASIA rat-neuron dataset and the virtually simulated dataset, and the download link is https://github.com/thomasaimondy/neuromorpho_neuron_type_classification.

**Figure 3:**
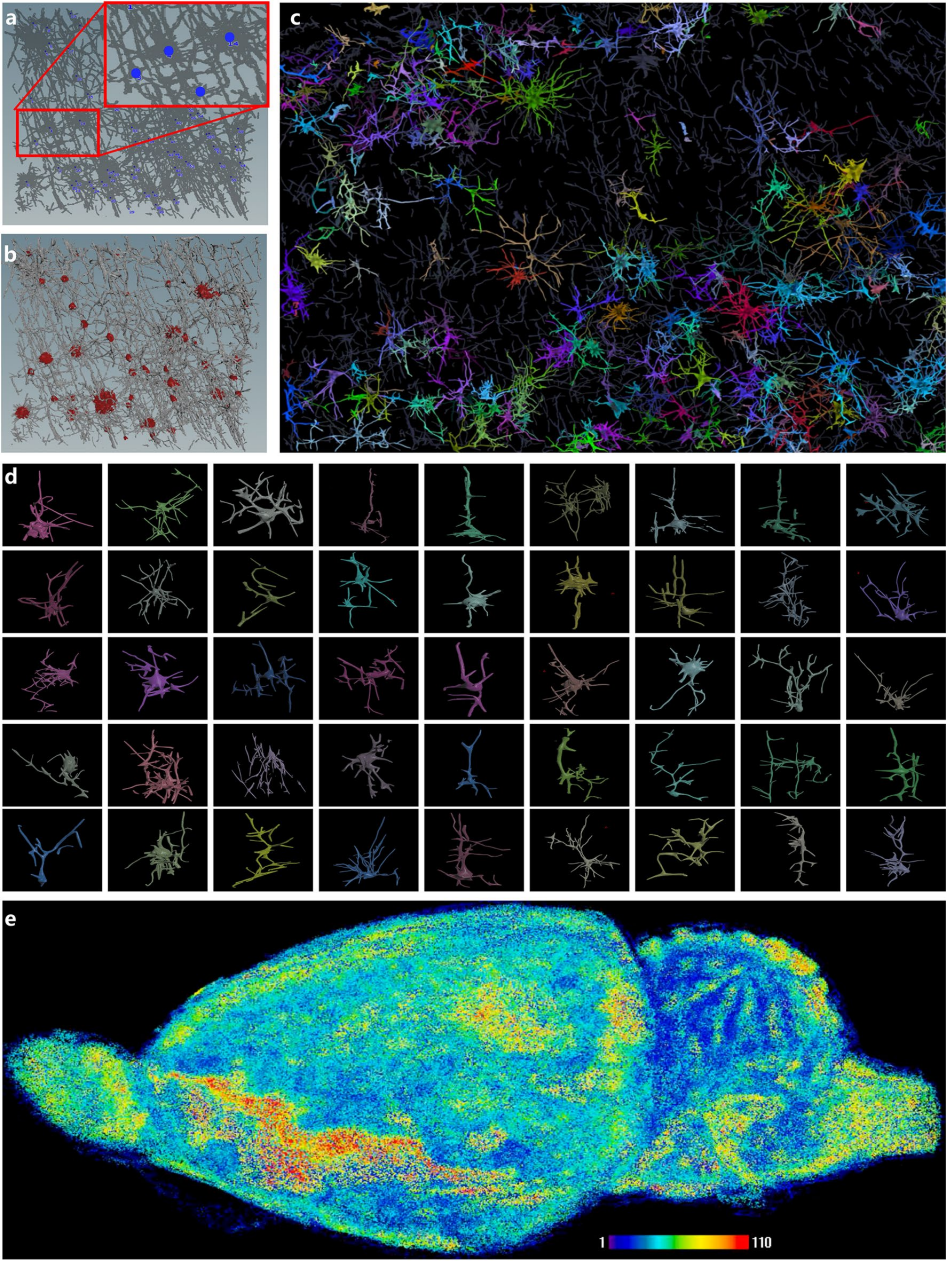
The self-reconstructed 2.6 million rat cells without labels. (**a**) Neuron branch detection and reconstruction. (**b**) Neuron soma identification and surface detection. (**c**) Different types of neurons were reconstructed from the neocortex; different colors represent different types of neurons. (**d**) The reconstructed CASIA rat-neuron dataset with approximately 2.6 million cells from a single rat brain. (**e**) The density of neurons in the whole rat brain.

Figure 3 shows the reconstruction procedure of the neuronal morphology. Figure 3a shows the reconstructed structures include somas and synapses, presented in gray, where each blue point represents the center position of soma, and the number nearby is the index of the soma. Both somas and synapses have a corresponding 3D position (e.g., X, Y, Z positions) and 3D size (e.g., the soma's radius in different directions). Figure 3b describes the reconstructed somas clustered based on the soma positions and their neighborhood pixels. Figure 3c shows the reconstruction and separation of each neuron in the area of the prefrontal cortex. The region size is 1300 × 1000 × 100 pixels and contains 100 neurons of different types. Some standard pyramidal cells and basket cells with morphological characteristics can be easily identified. Figure 3d shows some samples of the reconstructed CASIA rat neurons randomly selected from 2.6 million cells in a single rat brain. The morphologies of the neurons vary greatly, which shows the great challenge of their classification. Figure 3e shows the reconstructed whole rat brain, in which different colors represent different densities of the number of neurons.

### Neuron type classification

We tested the different classification models on three datasets: the virtually simulated dataset, NeuroMorpho-rat dataset, and CASIA rat-neuron dataset. Each sample in each dataset contains both three 2D images and a matched SWC-format file. The hand-designed features have also been calculated for these samples. There is a total of 31,501 raw images and 34,296 SWC-format files with labels. The sample ratio of training, validation, and test samples is 8:1:1. For the better training of DNNs, we expanded only the training samples, including 37,510 images (by translation, rotation, and scaling) and 40,196 SWC-format files (by adding or deleting some branches with Trees toolbox) in the 2-class task; and also 98,700 images and 99,996 SWC-format files in the 12-class task.

We tested the CNN model on 2D image samples and tested the Tree-RNN and standard RNN on the SWC-format dataset. The features in the hidden layers of the CNN and RNN are considered stable features from different data sources, and they are then integrated (by connecting two feature vectors) for the classification of the DNN model. The hand-designed features are classified with the support vector machine (SVM) model, which serves as the comparison experiment. The loss of SVM is L2-norm, and the stop-learning error is 1e-3.

For the virtually simulated dataset, basket and pyramidal cells are selected out with obvious morphology differences as the two classes of simple samples. The proposed models show 100% test classification accuracy on the virtually simulated dataset with two classes, including the proposed CNN, RNN, and standard SVM models.

**Figure 4:**
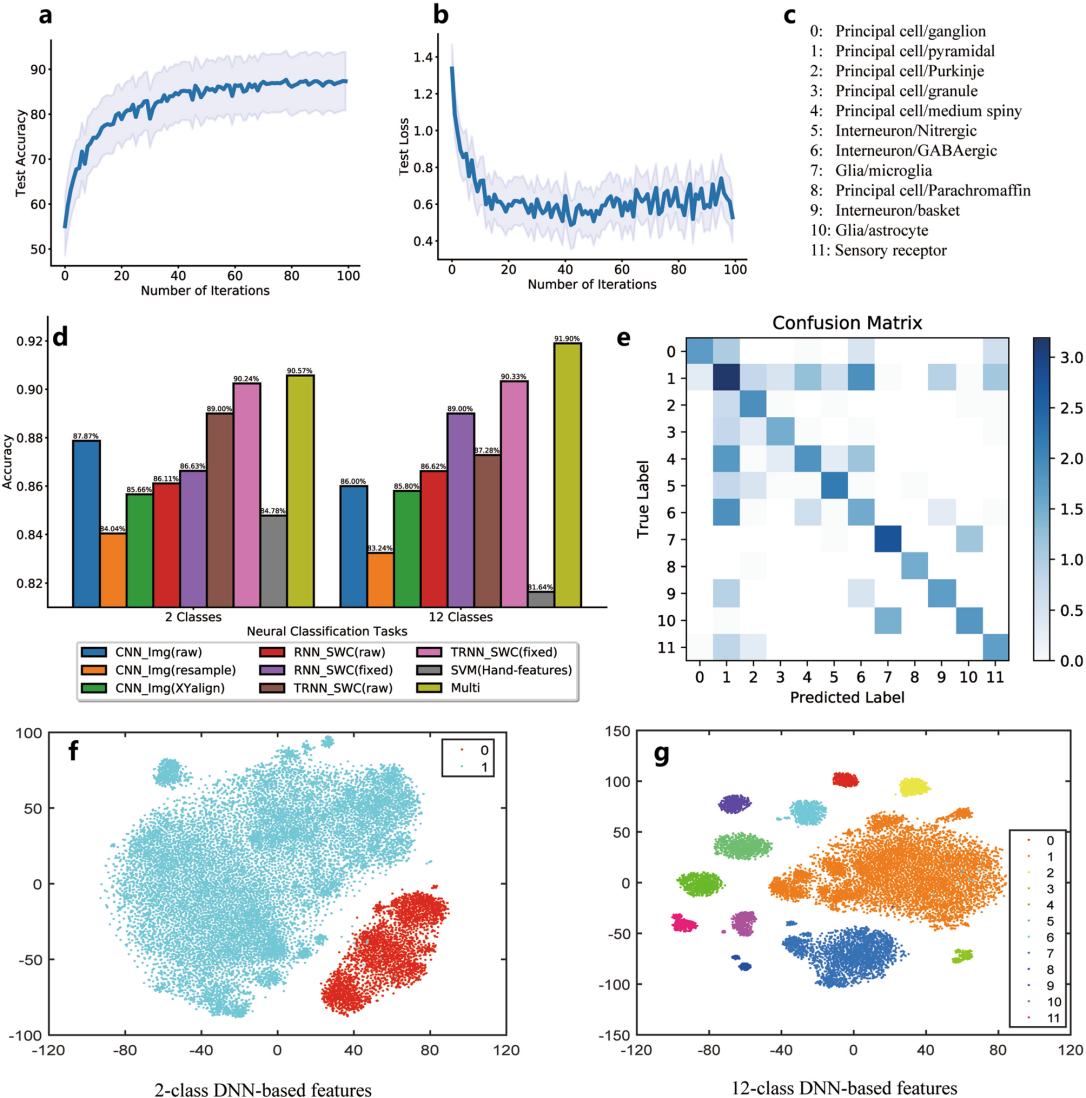
(**a**) The test accuracy with the number of iterations in the learning procedure of DNN-based neuron classification. (**b**) The loss curves in the test procedure of the DNN-based model. (**c**) The IDs and names of 12 subclasses of neuron types. (**d**) The performance of DNN-based models and other compared models on different types of datasets. (**e**) The confusion matrix, which shows the error classification in the 12-class identification. (**f**) The t-SNE distribution for the 2-class DNN-based features. (**g**) The t-SNE distribution for the 12-class DNN-based features.

For the NeuroMorpho-rat dataset, during the learning procedure in the DNN for the 12-class neuron type classification, the test accuracy is calculated as in Fig. 4a. The x-axis is the number of iterations. Here, we set this number to 100 for simplicity. The model in this figure integrates both the CNN and RNN features well for better classification performance. As the training time goes by, the test accuracy increases quickly, and the curve finally stops at approximately 90% accuracy.

The loss cost is another indicator of the learning procedure of the DNN model. As shown in Fig. 4b, the loss curve will decrease as the number of learning iterations increases, and at approximately 40 iterations, it obtains the smallest test loss; however, this is not the global minimal tuning point, and the test accuracy is still low. Hence, the network will continue training until it stops at the maximum number of iterations (100) with the dropout principle for anti-overfitting. All of the algorithms are run in a standard desktop computer with 4 GPUs (Tesla K40). The computation time for SVM is around 10 min; for RNN is around 2–6 h; for CNN is around 6 h; for Tree-RNN, it is around 24 h.

Figure 4c shows the IDs and the selected 12-type neuron names in the NeuroMorpho-rat dataset. As shown in Fig. 4d, in a two-class classification task, in which the two neuron types are principle cells and intern cells, for the single classification module, the RNN-based method obtains the best performances (90.24% accuracy for the Tree-RNN, and 86.11% and 86.63% accuracies for the standard RNN on raw SWC and fixed SWC data, respectively). Compared with them, the CNN methods obtain accuracies of approximately 84.04% to 87.87% based on the different qualities of the preprocessed image datasets (e.g., the raw SWC file, the resampled SWC files, and the XY-aligned SWC files). The SVM method for the classification of the hand-designed features achieves the lowest accuracy (84.78%). Then, we integrate the feature vectors of the CNN and RNN and obtain a 90.57% accuracy (with the “Multi” label in the figure).

There are six types of principal cells in the twelve-class classification task, three types of interneuron neurons, 2 types of glial cells, and 1 type of sensory receptor cell. Here, we can obtain a similar conclusion as for the two classes. For the single classification module, the best performance is that of the Tree-RNN on the SWC-format files, with a 90.33% accuracy; the CNN-based methods are approximately 83.24% to 86% accurate, and the SVM method is 81.64% accurate. Compared with these results, the proposed integrative method reaches a 91.90% accuracy, which is the highest and state-of-the-art performance on the NeuroMorpho-rat dataset.

Figure 4e shows the classification matrix for the CNN model on the 12-class neuron images. Each value in the matrix represents the number of target-classified labels ($N$) after the conversion of $log_{10} N$. The results show that some of the neuron types are more highly correlated; for example, the neuron morphology in class “1” is closer to that in classes “4”, “6”, “9” and “11”, and class “4” is highly connected to class “6”. Hence, misclassification usually occurs in these specific neuron types. This result shows that the different classes of neurons may share similar features, which shows the characteristics of the data's inner class and provides inspiration for better neuron-type assignments.

### t-SNE analysis on DNN-based features

DNN features for most of the neurons have hundreds of dimensions, for example, 512 for CNN and 128 for Tree-RNN, even the hand-designed features have 19 dimensions, which is much higher than that of the human beings might well perceive and understand. Here, we select t-SNE^32^ for the in-depth analysis of the distributions of high-dimensional features. The t-SNE can decrease the dimensions of the features to two or three dimensions, by which humans might easily obtain a better understanding.

Figure 4f shows the t-SNE analysis resulted from the 2-class DNN-based features from both the 2D-images and SWC-format files from the NeuroMorpho-rat dataset. There are two classes: “0” is for the principal cell, and “1” is for the interneuron cell. The initial data vector before t-SNE is 640 dimensions (with the integration of 512 from the CNN and 128 from the RNN). In this figure, the different cell types are separate, and only a few interneurons are miscategorized as principal cells. This result shows that CNN is powerful in feature detection. Figure 4g shows the t-SNE distribution of 35,000 rat neurons in 12 classes. Different classes of neurons can be categorized into different groups of samples. This means that the categorization of neuron types fits the characteristics of features learned from both the CNN and RNN.

### Analysis of hand-designed features

It is a long history for the hand-designed neuronal features that might contribute to the neuron-type classification to some scale. However, even the neurons in the same class are largely diverse, making it a hard problem for identifying neuron types given some limited features. Table 1 shows a collection of hand-designed neuronal features defined by the previous studies, including 19 features from morphology. For example, it contains “TotalLength” that indicates the summation of the length of all parts of neuronal structures (e.g., length of soma, dendrites, and axons), “MaxBranchOrder” introduces the maximum number of branch orders from the soma to terminals (axons or dendrites), and “SomaSurface” shows the total surface area of the soma.

**Table 1:** The hand-designed features related to the neuron morphology after determining the mathematical statistics.

Morphology Feature	Statistic Feature	Label
TotalLength	FractalDimension	NeuronName
TotalSurface	AverageDiameter	SpeciesName
TotalVolume	AverageContraction	PrimaryBrainRegion
TotalFragmentation	MaxBranchOrder	SecondaryBrainRegion
OverallDepth	MaxPathDistance	TertiaryBrainRegion
OverallHeight	MaxEuclideanDistance	PrimaryCellClass
OverallWidth	SomaSurface	SecondaryCellClass
NumberBifurcations	PartitionAsymmetry	TertiaryCellClass
NumberStems	AverageBifurcationAngleRemote	SpeciesAge
NumberBranches	AverageBifurcationAngleLocal	DevelopmentType

SVM is one of the most commonly used pattern-recognition algorithms. Here, we select it as the baseline classifier to further analyze these hand-designed features in Table 1. Not all of the features contribute to identifying the selected 12 neuron types with the SVM classifier. This phenomenon is not surprised for the limited numbers of features and neuron types.

**Figure 5:**
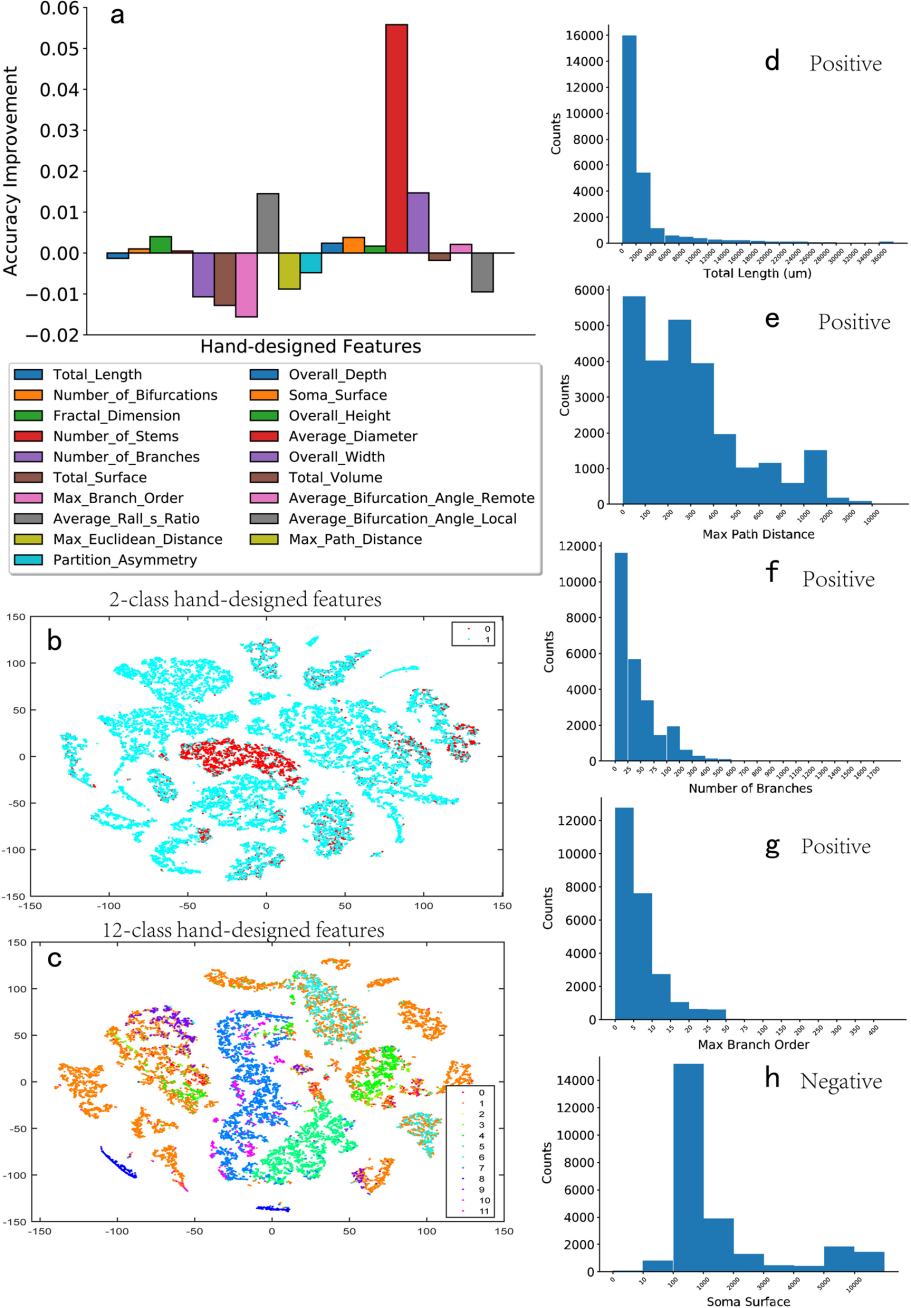
(**a**) Accuracy improvements of various hand-designed features. (**b**) The t-SNE distribution for the 2-class hand-designed features. (**c**) The t-SNE distribution for the 12-class hand-designed features. (**d**, **e**, **f**, **g**) Some positive hand-designed features for neuron type classification, including the total length, max path distance, number of branches, and max branch order. (h) Negative hand-designed features.

Further analysis is given in Fig. 5a, where contributions of different hand-designed features for the neuron-type classification are tested. From the figure, with the SVM classifier, we find that some features positively contribute to the classification performance, for example, the average diameter and overall width. In contrast, some others harm the accuracy, for example, the number of branches, the total surface, and the max branch order. This result indicates that some definitions of neuronal features by humans might be ineffective for these 12-type neurons. Additionally, the distributions of features in Fig. 5, to some extent, show the importance of different features.

Figure 5d shows the total-length feature, in which the minimum length in it is tens of micrometers, and the maximum length is 36,000 µm. The y-axis is the count of the neurons at different length scales. The curve's distribution is the power-law distribution, which shows that more of the neurons have local-type connections instead of long-term-type connections.

Figure 5e shows the max path distance feature, and the range of the size is from 0 to 10,000. Most neurons are in two ranges: from 0 to 100 µm and from 200 to 300 µm. The count number of the max path distance exhibits linear attenuation with the distance.

Figure 5f shows the number of branches feature, in which the count number of neurons dramatically decreases with the increase of the number of branches. The total number of branches ranges from 0 to 1,700, which means that the maximum number of branches in a neuron can reach 1,700, i.e., a large number for most traditional neurons. However, most neurons stay in a range of branch orders from 0 to 300, following a power-law-like distribution.

Figure 5g shows the max branch order, which is another positive hand-designed feature for neuron type classification. However, Fig. 5h shows a negative feature, i.e., the soma surface, ranging from 0 to 10,000. The soma surface contributes little to the neuron classification instead of well accepted as an important neuron type feature. It also follows a normal-like distribution, very different from the power-law distribution of other features. Further analysis of what kind of feature distribution will contribute to the classification is a next-step candidate key research point.

### t-SNE analysis of hand-designed features

Figures 5b and c show the t-SNE analysis results for the hand-designed features with a 19-dimensional vector on two classes and 12 classes, respectively. The hand-designed features are mostly from anatomical experiments or the simple statistics of neuron characteristics, which may not be the best indicator for neuron types. The comparisons between DNN-based features (Figs. 4f and g) and hand-designed features (Figs. 5b and c) show that the DNN-based features have better clustering performance. Hence, they will be better for the neuron type indicator in neuron type classification and categorization.

## Discussion

Designing the proper morphological features for neuron categorization and classifying them are the two main challenges. Some traditional methods use hand-designed features and shallow network models for the neuron type classification. However, the morphologies of neurons are complicated and are usually described as raw 3D images with billions of voxels or SWC-format files with unstructured data lengths. These data characteristics cause the traditional machine learning methods to fail. Different types of DNN-based efforts have solved these problems to some scale, especially in brain-tissue segmentation^33^, tracing^34^, and classification^35^.

This paper, similar but different from other DNN-based algorithms, proposed an integrative deep learning model for better neuron-feature generation and neuron-type classification. The model contains a CNN module for feature detection in structured 2D images via the axes' projection from 3D raw images. We also self-designed a proper tree-based RNN for unstructured feature learning from SWC-format files.

Compared with the traditional hand-designed neuron features (e.g., the size of the soma and the number of branches), the DNN-based features have a more extended dimensional representation. For example, they contain a 512-dimensional CNN feature vector and a 128-dimensional Tree-RNN feature vector. Moreover, the DNN-based feature has better clustering characteristics in the t-SNE distribution. These neuron morphology representations may contribute to a finer neuron categorization and classification for the whole rat brain. The CNN model is pre-trained on the CASIA-rat dataset and then integrates with Tree-RNN to retrain and test virtual simulated and NeuroMorpho-rat datasets. The integrated model achieves the best performance compared with traditional SVM, CNN, and RNN models on the standard NeuroMorpho-rat dataset.

The structural and functional identifications of different neural types and related network topologies are important for the next-step research on biology-inspired artificial intelligence. Yin et al. have analyzed degree centrality, closeness centrality, and betweenness centrality of conventional and clustered networks to better understand the biological efforts to optimize the network information transfer^36^. Hence, deeper integration of these multi-scale biologically-plausible inspirations with artificial or spiking neural networks is necessary towards brain-inspired artificial intelligence.

## Methods

**Figure 6:**
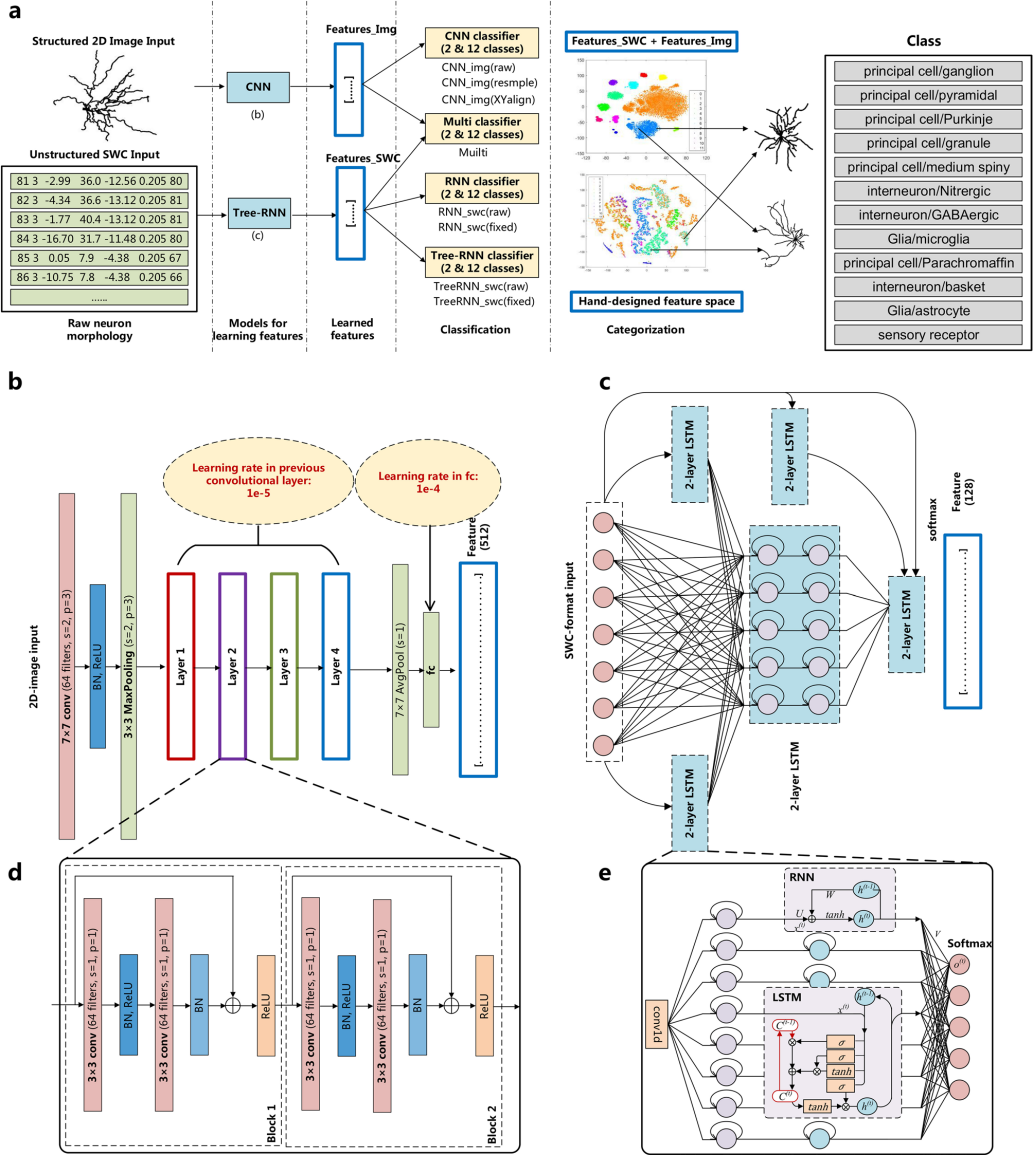
The proposed integrated DNN architecture contains a ResNet CNN for 2D-image feature detection and Tree-RNN for SWC-format feature detection. (**a**) Diagram of the processing procedure, including the data preparation, feature learning, neuron classification, and categorization. (**b**) The structure of ResNet18 for image classification. The input is three-channel images from the projections of the X, Y, and Z axes. Before the residual blocks, data are first processed by convolution, batch normalization (BN), ReLU activation, and MaxPooling. The following four layers have different residual block hyperparameters, as shown in Table 2. The output is the generated features after completing average-pooling and full-connection. (**c**) The proposed Tree-RNN, including the standard structures of the RNN and LSTM modules. There are five 2-layer LSTM blocks in this tree, and black arrows represent the connections between them. Every block has the same structure, which contains two hidden layers, each with 128 neurons. Finally, the result is output through a fully connected layer. The dotted boxes show the basic operating units of the RNN and LSTM. (**d**) The submodule of the ResNet layer. (**e**) The submodule of the Tree-RNN, which can also be considered the traditional simple 2-layer RNN.

The overall processing procedure is shown in Fig. 6a, which is designed to answer the following three basic questions related to neuron classification and categorization:

*Which are the most proper models for different neural data formats?* The neuron morphology community usually uses tree-like SWC-format data and raw 3D pixel data for the morphology description. However, SWC-format data have variable lengths related to the complexity of structures, and 3D data may contain millions of pixels, which is a big data problem for the next-step classification. Based on these two challenges, we select the RNN and Tree-RNN to classify variable-length SWC-format data and select the ResNet CNN to classify 2D images (converted from 3D raw images by the projection of the X, Y, and Z axes), as shown in the models for learning features in Fig. 6a.

*What level of granularity of a cell type definition is necessary?* Different scales of neuron classification are considered in the classification in Fig. 6a. For the large scale, two neuron types are classified, including principle cells and intern cells. For the small scale, 12 sub-neuron types are classified, including six types of principal cells (e.g., ganglion, granule, medium spiny, parachromaffin, Purkinje, and pyramidal cells), three types of interneurons (e.g., basket, GABAergic, and nitrergic cells), two types of glial cells (e.g., microglia and astrocyte cells) and 1 type of sensory receptor cell.

*What are the fundamental features guiding the categorization of various cell types?* Usually, people use hand-designed features after a careful selection, for example, the size of the soma, the number of branches, the angle of bifurcation, the branch level, the branching order, the neuron length, the dendritic shapes, or the spine density. However, these features are too simple compared with the far more complex neuron types. The DNN-based feature learned from both SWC-format and image-format data may give us more hints about the proper definition of features, as shown in the categorization in Fig. 6a.

### ResNet CNN for the 2D-morphology image classification

As shown in Fig. 6b (a more detailed version is shown in Appendix Fig. 1), for the classification of neuron images, we construct a ResNet (residual neural network)^37^. The residual blocks in the ResNet (i.e., the dotted box in Fig. 6d) are carefully designed for the vanishing gradient problem. Compared with traditional DNNs, the ResNet protects the integrity of information and avoids gradient disappearance and gradient explosion, which makes it more powerful for 2D image classification. In addition, taking the complicated structural features of neuron morphology into consideration, we set the layer number of the ResNet to 18 and the learning rate to $10^{ - 5}$ for previous layers and $10^{ - 4}$ for the last fully connected layers. The remaining computational units are similar to those of the traditional CNN, and the detailed parameters of the CNN can be found in Table 2.

**Table 2:** The hyperparameters for the CNN, RNN, and Tree-RNN.

CNN	RNN	Tree-RNN
lr-fc = 1e-4	lr = 1e-4	lr = 1e-4
lr-conv = 1e-5	n-hidden = 128	n-hidden = 128
Epochs = 20	Epochs = 100	Epochs = 100
Batch-size = 300	Batch-size = 400	Batch-size = 500
Optimizer = Adam	Maxlen-of-tree = 1500	Maxlen-of-tree = 1500
Loss = CrossEntropyLoss	OverallHeight	in-dim = 7

### Tree-RNN for unstructured SWC-format data classification

SWC-format files (i.e., the initials of the last names of E.W. Stockley, H.V. Wheal, and H.M. Cole^38^) present the tree structure and describe the order of root nodes or branch nodes of neurons. They are non-structured and difficult to analyze further by traditional machine learning methods. In addition, the data in an SWC-format file also usually contain significant morphological errors, e.g., false mergers and false splits, which make the neuron type classification difficult.

Long short-term memory (LSTM)^39^ networks have gate mechanisms and are more powerful for variable spatial or temporal information processing. Here, we select both LSTM and traditional RNN units as the basic RNN module for morphology's unstructured feature learning. As shown in Fig. 6e, we use standard 2-layer LSTM^39^ and RNN modules (and LSTM) to classify unstructured SWC-format data.

To achieve a better classification, we design a convolutional layer for data dimension reduction and then integrate it into the recurrent module with the full connection. The number of hidden units of the RNN and LSTM is 128 for both of them. The output layer has 2 or 12 classes according to the experiments.

Furthermore, the neuron morphology in an SWC-format file describes the tree-type architectures of the neuron structures; hence, the “tree-like structure” will be valuable in the data organization of the SWC-format file. We then hand-design the “tree-structure-based RNN” (Tree-RNN), as shown in Fig. 6c, which connects different RNN modules by a basic tree structure. Each RNN module has a 2-layer LSTM (or RNN) and consists of two hidden layers (each with 128 neurons). The features in the Tree-RNN before the final fully-connected layers can also be combined with feature vectors in the CNN for integrative feature representation and classification (e.g., the “Multi” model). Detailed parameters of the RNN and Tree-RNN are given in Table 2. In addition, we also give further performance comparisons between different DNN models, as shown in Table 3, where accuracy and the area under the curve are tested^30,40–42^.

**Table 3:** The performance of neuron-type classification with different DNN architectures, including accuracy and the area under the curve (AUC).

Name	Architectures	Accuracy (%)	AUC
CNN^40^	ResNet	87.87	0.824
RNN^42^	2layer-LS™	89.00	0.882
Tree-RNN^30^	Tree-2layer-LS™	90.33	0.894
U-net (with a full-connection layer for classification)^41^	Unet	86.39	0.781
Ours (Combination last layer, lr=1e-3)	ResNet+TreeRNN	89.36	0.907
Ours (Combination last layer, lr=1e-4)	ResNet+TreeRNN	91.90	0.932
Ours (Combination last layer, lr=1e-5)	ResNet+TreeRNN	91.13	0.928
Ours (Combination hidden layer, lr=1e-3)	ResNet+TreeRNN	88.52	0.892
Ours (Combination hidden layer, lr=1e-4)	ResNet+TreeRNN	90.77	0.916
Ours (Combination hidden layer, lr=1e-5)	ResNet+TreeRNN	90.69	0.909

### The hand-designed features

The hand-designed features include both the features related to morphology (e.g., the length, surface size, width, depth, or the number of branches) and the features obtained after completing the mathematical calculations (e.g., the fractal dimension, the calculated surface area of the soma, and average bifurcation angles at remote or local scales), as shown in Table 1. The samples' labels cover different scales, from neuron types and brain regions to species and developmental characteristics. These features will be used for the feature-based SVM classification and the comparison analysis with DNN-based features in categorization.

### Trees toolbox for preprocessing SWC-format files

We use the Trees toolbox for neuron morphology generation, conversion, feature calculation, and the repair of SWC-format files^24^.

1 $$ \begin{array}{*{20}c} {\delta z = z_{min} } & {if(\delta z > Z_{th} )} \\ \end{array} $$

As shown in Eq. (1), ${\updelta }_{z}$ is the difference between the pre- and post-nodes at the Z-axis of an SWC-format file. $Z_{th}$ is the predefined hyperparameter (e.g., shown in Fig. 1b). Here, we set $z_{min}$ to zero.

### t-SNE configuration for feature analysis

The t-SNE (t-distributed stochastic neighbor embedding) is a nonlinear dimension-reduction algorithm for mining high-dimensional data^32^. Compared with the traditional linear dimension-reduction algorithm, i.e., PCA, which cannot explain complex polynomial relations between features and performs poorly when they focus on different data points in lower-dimensional regions, t-SNE preserves both the local and global structure of the data and is a very suitable dimension-reduction algorithm for the process of feature clustering.

2 $$ p_{j/i} = \frac{{exp( - ||x_{i}^{640d} - x_{j}^{640d} ||^{2} /2\sigma_{i}^{2} )}}{{\sum\limits_{k \ne i} e xp( - ||x_{i}^{640d} - x_{k}^{640d} ||^{2} /2\sigma_{i}^{2} )}} $$

For the DNN-based features, we use two feature points $x_{i}^{640d}$ and $x_{j}^{640d}$ (both of which have 640 dimensions) in the high-dimensional space and calculate the Gaussian distribution centered on $x_{i}^{640d}$ by $p_{ij} = \frac{{p_{j/i} + p_{i/j} }}{2n}$. $\sigma_{i}$ represents the variance and $n$ is the number of candidate points.

When mapping the data to low-dimensional space, we select the 2D space. We still need to reflect the similarity between high-dimensional and low-dimensional data points in the form of conditional probability $q_{ij}$.

3 $$ q_{ij} = \frac{{\left( {1 + ||y_{i}^{2d} - y_{j}^{2d} ||^{2} } \right)^{ - 1} }}{{\sum\limits_{k \ne l} {\left( {1 + ||y_{k}^{2d} - y_{l}^{2d} ||^{2} } \right)^{ - 1} } }} $$

We use the conditional probability distribution $P_{i}$ (in the low-dimensional space) and $Q_{i}$ (high-dimensional space) with the conditional probability between every two points to measure the similarity (Kullback–Leibler divergence) between these two distributions (as shown in Eq. (4)).

4 $$ C = KL(P||Q) = \sum\limits_{i} {\sum\limits_{j} {p_{ij} } } log\frac{{p_{ij} }}{{q_{ij} }} $$

The gradient is calculated by:

5 $$ \frac{\partial C}{{\partial y_{i}^{2d} }} = 4\sum\limits_{j} {\left( {p_{ij} - q_{ij} } \right)} \left( {y_{i}^{2d} - y_{j}^{2d} } \right)\left( {1 + \left\| {y_{i}^{2d} - y_{j}^{2d} } \right\|^{2} } \right)^{ - 1} $$

Then, the stochastic gradient descent algorithm is trained, and the best 2D clustering samples are obtained, which could be considered the best 2D information representation for the original 640D distribution.

## Supplementary Information

Supplementary Information

## Data Availability

The source code, three types of datasets (for the raw data of neuromorpho-rat, please downloading it directly from neuromorpho.org), and the trained DNN-based models are available https://github.com/thomasaimondy/neuromorpho_neuron_type_classification.
