# Supplementary material for: Neuron type classification in rat brain based on integrative convolutional and tree-based recurrent neural networks
Source: Sci Rep. 2021 Mar 31;11:7291. doi: 10.1038/s41598-021-86780-4 (PMC8012629; doi:10.1038/s41598-021-86780-4)
Supplement: Supplementary file 1 — Supplementary Information [file 41598_2021_86780_MOESM1_ESM.docx]

**Neuron Type Classification in Rat Brain Based on**

**Integrative Convolutional and Tree-Based Recurrent Neural Networks**

Tielin Zhang^1,*^, Yi Zeng^1,2,3,*^, Yue Zhang^4^, Xinhe Zhang^1^, Mengting Shi^1,2^, Likai Tang^5^ , Duzhen Zhang^1,2^ and Bo Xu^1,2,3^

^1^Institute of Automation, Chinese Academy of Sciences (CAS), Beijing, China

^2^University of Chinese Academy of Sciences, Beijing, China

^3^Center for Excellence in Brain Science and Intelligence Technology, Chinese Academy of Sciences, Shanghai, China

^4^Electronics and Communication Engineering, Peking University, Beijing, China

^5^Tsinghua University, Beijing, China

^*^**Corresponding authors**

E-mails: tielin.zhang@ia.ac.cn and yi.zeng@ia.ac.cn.

**Appendix Figure**


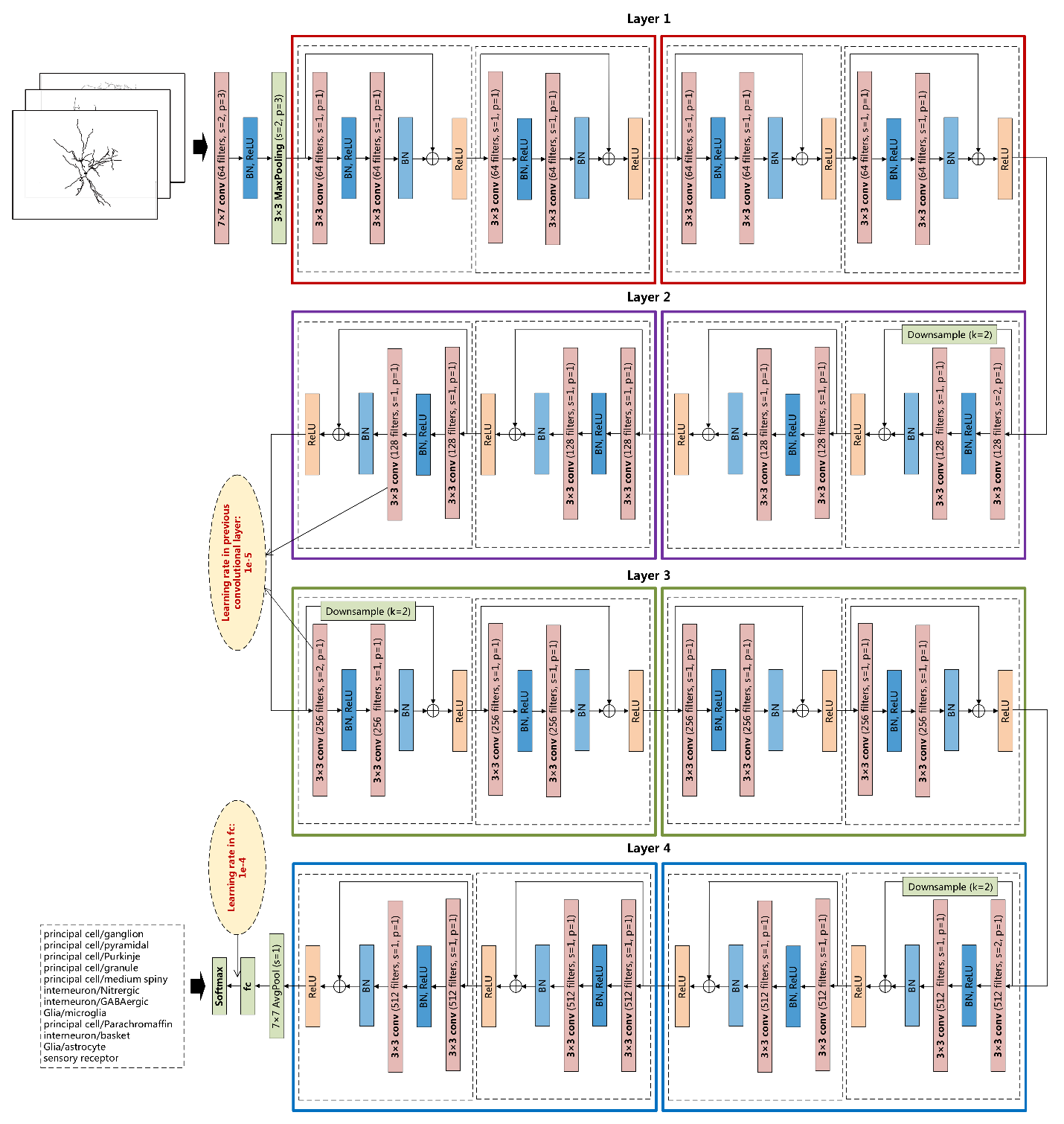


**Appendix Fig. 1.** The detailed architecture of CNN model.
